# The Effect of *Arthrospira platensis* Capsules on CD4 T-Cells and Antioxidative Capacity in a Randomized Pilot Study of Adult Women Infected with Human Immunodeficiency Virus Not under HAART in Yaoundé, Cameroon

**DOI:** 10.3390/nu6072973

**Published:** 2014-07-23

**Authors:** Frank Stéphane Winter, Francois Emakam, Anfumbom Kfutwah, Johannes Hermann, Marcel Azabji-Kenfack, Michael B. Krawinkel

**Affiliations:** 1 Institute of Nutritional Sciences, University of Giessen, Giessen D-35392, Germany; E-Mails: Frank.Winter@ernaehrung.uni-giessen.de (F.S.W.); ndooh@hotmail.com (F.E.); Johannes.Herrmann@ernaehrung.uni-giessen.de (J.H.); 2 Laboratory of Virology, Centre Pasteur Cameroun, Yaoundé 00237, Cameroon; E-Mail: Kfutwah@pasteur-yaounde.org; 3 Faculty of Medicine and Biomedical Sciences, University of Yaoundé I, Yaoundé 00237, Cameroon; E-Mail: azabji@gmail.com

**Keywords:** HIV, pre-HAART, supplement, *Spirulina*, antioxidant

## Abstract

Dietary supplements are often used to improve the nutritional status of people living with HIV/AIDS (PLHIV). *Arthrospira platensis* (*Asp*), also known as *Spirulina*, is a cyanobacterium rich in proteins and micronutrients. Cell and animal trials described immune-modulating, antiretroviral and antioxidant activities. This pilot study describes the effects of the supplementation of 5 g/day of *Asp* on a pre-highly-active antiretroviral therapy (pre-HAART), HIV-infected, adult female population. It was conducted as a three-month randomized controlled trial (RCT) that compared a cup supplementation of five grams/day of *Asp* with a placebo of equal protein content and energy. The study included 73 HIV-infected women. The immediate outcome variables were CD4 T-cells, viral load and immune activation by CD8 T-cells expressing CD38. The antioxidant status was assessed by way of the total antioxidant capacity of the serum (TAOS). The renal function was documented by way of creatinine, urea and the calculated glomerular filtration rate. Statistical analyses were carried out with non-parametric tests, and the effect size of each interaction was calculated. No differences in the immunological and virological markers between the *Asp* and the placebo group could be observed. In the placebo group, 21 of 30 patients (70%) developed concomitant events, while in the *Asp* group, only 12 of 28 patients (43%) did. Both groups registered a significant weight increase; 0.5 kg (*p* < 0.05) in the *Asp* group and 0.65 kg (*p* < 0.05) in the placebo group. The antioxidant capacity increase of 56 (1–98) µM for *Asp* was significantly different from the decrease observed in the placebo group (*p* < 0.001). A slight increase in the creatinine level of 0.1 g/dL (*p* < 0.001) was observed in the *Asp* group, and no effect was observed in the urea levels. The improvement of the antioxidant capacity under *Asp*, shown for the first time on PLHIV, could become a focus for future research on the nutritional and health effects of *Spirulina*. The observed slight, but significant increase of serum creatinine needs further evaluation, especially with varying doses of *Asp*.

## 1. Introduction

At present, it is estimated that 34 million (range: 31.4–35.9 million) people worldwide are living with HIV, of whom, 23.5 (22.1–24.8) million are living in Sub-Saharan Africa [1,2]. The life expectancy of people living with HIV (PLHIV) has greatly improved due to the availability of highly-active antiretroviral therapy (HAART) [3]. However, only 36% of people in need of treatment in Sub-Saharan Africa have access to it [4].

This study was carried out in Cameroon, a country with an HIV prevalence of 4.3%, considered as one of the highest in Western and Central Africa [5]. The majority of PLHIV are found in the two major cities of the country, which make up about 20% of the total population. In Yaoundé, 8.8% of women and 3.9% of men are HIV infected [6]. Access to care has considerably increased since 2007 and reached 41% countrywide in 2010 [7].

One major problem related to HIV-infection, especially in Sub-Saharan Africa, is malnutrition [8]. HIV and undernutrition negatively affect each other in a vicious circle [9]. In particular, the deficiency of vitamins, minerals and antioxidants plays a critical role in the progress of HIV [10]. However, a review of high-dose micronutrient trials has not shown sufficient evidence to revise the WHO recommendation endorsing an intake at generally recommended dietary amounts [11]. The implementation of dietary support programs has been successful in strengthening medical care and its health outcomes [12,13,14]. Complementary to the essential role of nutrition, some aliments presented functional capacities with regard to immunity and detoxification. For example, a major topic for an efficient immune system in the case of a chronic disease burden is the sustainment of a high level of antioxidants [15,16].

The current trend for HIV and other chronic diseases is personalized nutrition [17]. Sustainable nutrition is based on knowledge and on the implementation of local products to cover daily dietary needs. Besides the nutritional aspect, aliments containing bioactive agents offer a great opportunity to have an impact on the betterment of health. In particular, the rich therapeutic plant diversity in Africa could enhance the health of people in need [18,19].

In Durban in 2005, international agencies and national health departments of African States defined the role of nutrition in the fight against HIV in Africa. The fourth point of their statement has been one of the motivations for this study; “there is a proliferation in the market place of unproven diets and dietary therapies, with exploitation of fears, raising false hopes, and further impoverishment of those infected and affected by HIV and AIDS” [20].

Dietary interventions aiming at a delay of the progression to AIDS must focus on body weight, antioxidant status, viral load and/or CD4 count to describe potential changes in disease progression [21].

*Arthrospira platensis* (*Asp*) contains a rich diversity of bioactive molecules displaying a potential to improve the health of people [22,23,24,25]. Cyanobacteria are already available and considered as a safe dietary supplement [26]. Cell culture and animal studies with *Spirulina* have described antioxidant and antiretroviral activity, as well as an enhancement of immune defense [27,28,29]. Since the algae have been proposed to have antiretroviral activity, more research is required in order to clarify their effect and implementation [30,31].

The present study examined the effects of an intake of five grams per day of *Arthrospira platensis* focusing on the antiretroviral effect, immune function, antioxidative potential and renal function. The intervention added 21 kcal covering about 1% of the daily energy need. The protein supplement of the intervention was three grams, representing 6% of the daily protein need of women.

## 2. Experimental Section

The study was a three-month pilot, randomized, double-blind and placebo-controlled intervention. It was conducted according to the guidelines laid down in the Declaration of Helsinki, and all procedures involving human subjects were approved by ethical clearance through the ethical board of the faculty of medicine of Giessen University, Germany, and the national ethics committee of Cameroon (No. 131/CNE/SE/2010, July 16, 2010, Yaoundé). It was registered at ClinicalTrials.gov (NCT01084382). Informed consent was obtained from all subjects under the guidelines established.

The study population were HIV-infected, HAART-naive women receiving outpatient care in health centers of Yaounde, Cameroon, between June and December, 2010. The women enrolled were not pregnant or breast feeding, had a BMI below 26 kg/m^2^ and a CD4 T-cell count of <600 CD4 cells/mm^3^. The randomization into two groups was achieved chronologically for the persons recruited by using a randomly generated MS Excel^®^ list dividing the participants into two groups with an equal number of patients.

The product under investigation was 100% *Arthrospira platensis* powder provided by EARTHRISE^®^ Nutritionals (Irvine, CA 92612, USA). The placebo contained the same amount of protein and energy using pea protein isolate Pisane F9 mixed with Dextrans (EMDEX™, HERBAMED GmbH, D-53881 Euskirchen, Germany). The composition of both supplements are presented in Table 1. An amount of five grams was administered in ten pills per day with 500 mg in each tablet. This represents an acceptable amount of pills per day. There was no difference between the placebo and the *Asp* supplement regarding appearance and taste. The blister had an ID number without significance for the patient. This ID had been translated into A and B supplements for optimal handling and organization of the distribution. The group attribution was cleared after the database was controlled and closed for analyss. The blinding was achieved by identical packaging provided by “greenValley Naturprodukte” GmbH (D-10997 Berlin, Germany).

**Table 1:** Nutritional composition of the intervention and placebo products.

Nutritional Composition	*A. platensis* (500 mg)	Placebo (500 mg)
Energy	8.9 kJ/2.1 kcal	8.8 kJ/ 2.1 kcal
Protein	310 mg	310 mg
Carbohydrate	77.5 mg	186 mg
Lipid	35.5 mg	4 mg

Compliance was assessed in a monthly meeting between the patients and the physician in charge. The discussion was based on documentation filled in by the patients showing the number of pills and concomitant events for each day. The proposed concomitant events were described as: anorexia, fatigue, nausea/vomiting, dry or productive cough, abdominal pain, diarrhea and constipation. The grading was defined as “not at all”, “a little”, “as usual”, “a lot” and “enormous”. The physician filled in the clinical registry in which concomitant events were registered with the dates of the beginning and the end and the actions undertaken.

The consultation was combined with the distribution of the supplements and the refund of the transportation charge.

### 2.1. Marker Variables

The blood samples were collected at the out-patient unit of the Central Hospital and brought for analysis to the nearby Centre Pasteur laboratory at baseline and after 12 weeks. The samples were collected between 10 a.m. and 12 p.m. The same materials and workflow were respected for all samples.

A CD4 T lymphocyte marker was measured by way of FACS-count^®^ (Becton, Dickinson and Company, Franklin Lakes, New Jersey 07417-1880, USA).

The viral RNA was extracted from the plasma with the QIAamp^®^ Viral RNA Mini extraction kit (Qiagen Inc., Chatsworth, CA, USA). The plasma viral load was run with an RT-PCR kit, known as Generic HIV charge Viral^®^ (Biocentric, F- 83150 Bandol, France). All patients with an undetectable viral load were screened with an ELISA for the different variants of HIV-1 and HIV-2.

The measurement of the CD38 antigen expression was performed on the Coulter^®^ Epics XL/XL-MCL with the antibody combination: PE-CD3 and PE/Cy5-CD and florescent isothiocyanate, FITC-CD38, from (eBioscience Inc., San Diego, CA 92121, USA)

Body weight was assessed monthly with a digital scale to the nearest 0.1 kg with the participants barefoot and wearing light clothes only (seca, D-22089 Hamburg, Germany). Height was measured with a standing board scaled to the nearest 0.5 cm. BMI (kg/m^2^) was calculated from weight (kg) and height (m).

The antioxidant potential was measured as the total antioxidant status in blood serum (TAOS) by the Trolox Equivalent Antioxidant Capacity Assay kit CS790^®^ (Sigma-Aldrich, St. Louis, MO 63103, USA) [32].

The analyses of the renal function blood variables were performed using the system, Vitros 250^®^. The eGFR was calculated with the modification of the diet renal disease (MDRD) formula [33], adjusted for African women.

### 2.2. Statistics

The main objective was to determine whether the effects of a three-month supplementation of five grams of *Arthrospira platensis* were superior to a placebo regarding the course of the disease.

The variables were described by the change occurring during the intervention with the median, inter-quartile range. The expected superiority of *Asp* over the placebo was checked by testing the null hypothesis. As the cohort size was around or less than 30 patients and in order to achieve a robust analysis, the non-parametric Mann–Whitney *U*-test and the Wilcoxon rank tests were applied. The effect magnitude of all statistic tests performed was reported by the effect size “r”. The correlations were achieved via the Spearman coefficient. All statistical calculations were performed with SPSS version 20.0 (SPSS, Chicago, IL, USA).

## 3. Results

### 3.1. Population

**Scheme 1:**
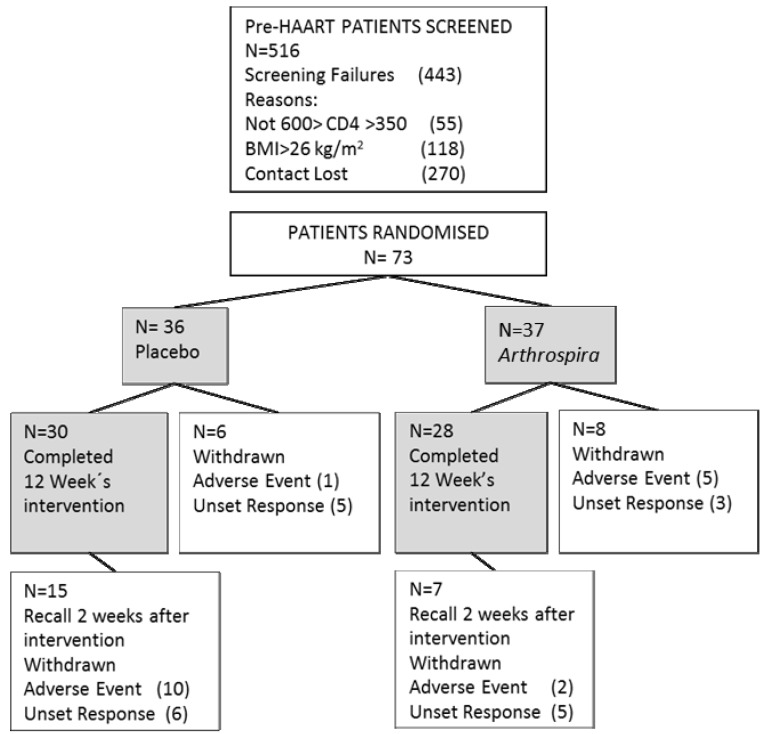
Participant flow in the course of the study. HAART, highly-active antiretroviral therapy.

From a screened population of 513 patients from three different health centers in Yaoundé, 73 patients were recruited between May and July, 2010 (see Scheme 1).

The intervention compliance was 78%. The major reason for drop-outs during the intervention was the “failure to follow up”, with 50% of all drop-outs. The compliance was also affected by the health need of patients to start HAART. Only three patients started an antiretroviral therapy during the intervention, but ten of the placebo patients and two patients of the *Asp* groups started HAART in the two weeks after the intervention. The total of those starting HAART was 15 patients, which represented a rate of 20% of the total population and 41% of the total drop-out patients. The difference of those starting HAART across the groups presented a non-significantly higher proportion in the placebo group than in the *Asp* group. Furthermore, under placebo, 21 out of 30 patients (70%) developed one of the following concomitant events: opportunistic infections, diarrhea, headache, fatigue, respiratory disease or gastrointestinal symptoms. This rate was, in the *Asp* group, only 12 of 28 patients (43%). The major event in both groups with a 30% incidence was malaria. Furthermore, the groups differed in asthenia and cephalic, with five cases in placebo and two in *Asp*.

The 58 patients who enrolled and completed the intervention were unmarried, had at least a primary education and were of a median age of 32 (23–35) years old, showed no signs of wasting syndrome, had a median CD4 count of 441 (346–489) cells/mm^3^ and a BMI of 22 (20–23) kg/m^2^. The dietary diversity consumed over the study did not change and stayed constant for all patients at a score of four dietary groups per day.

The first outcome of the intervention was a non-significantly higher proportion of patients starting HAART in the placebo than the *Asp* group, ten and five patients, respectively. There was a higher incidence of concomitant events, 70% in the placebo group *versus* 43% in the *Asp* group. With these observations, it could be useful for future studies to assess the properties of *Spirulina* in relation to the time extent from seroconversion to initiation of HAART.

### 3.2. Blood Analyses

**Figure 1:**
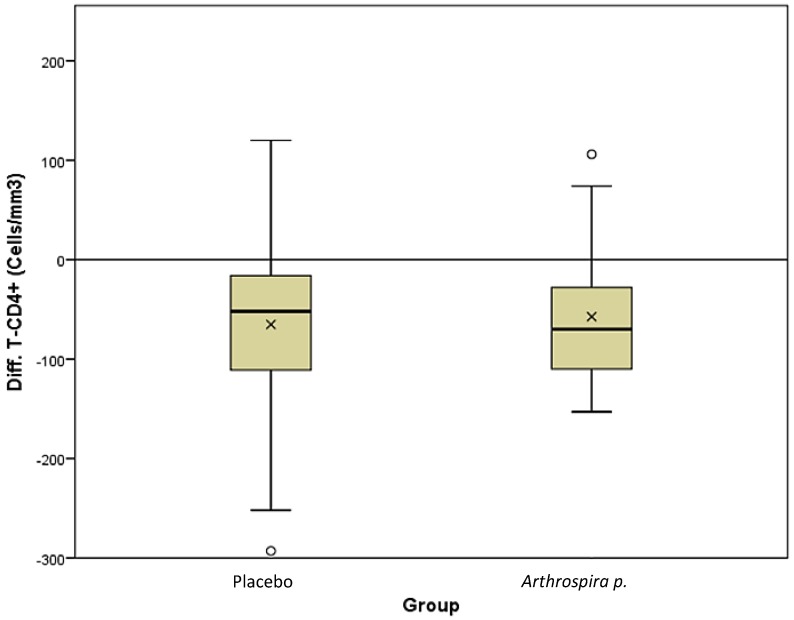
The difference (Diff) of the CD4 lymphocyte counts between the groups receiving placebo and *A. platensis* at the end of the study. The box plot represents the median and the IQR. The zero line represents no change. The crosses indicate the means.

As mentioned above, the study objective is documented by way of the CD4 T-cell count difference between the two products during the pilot RCT. The non-parametric comparison showed no difference between the groups in the variable progression over three months, *r* = 0.01, *p* = 0.926 (see Figure 1). Both groups presented a significant decrease: for placebo, by −52 (−111.8–−16.5) cells/mm^3^, *r* = 0.67, *p* < 0.001; and for *Asp*, by −66 (−111.5–−20.5) cells/mm^3^, *r* = 0.61, *p* < 0.001. An effect size above 0.5 stands for a large effect, and it has to be assumed that the decrease concerns most of the involved patients in both groups.

The viral load (VL) quantification method was restricted to HIV type 1 viruses. In two patients, the virus was undetectable. HIV-2 viral load was not systematic. However, serological tests revealed two patients, one in each group, with an HIV type 2 infection. The study did not test for double infections with HIV-1 and HIV-2. As can be expected in pre-HAART patients presenting an advanced infection [34], the VLs at baseline were high, with 5.5 (4.3–6) log10 corresponding to 315,000 (20,000–1,000,000) copies/mL. The HIV-1 load did not significantly change between the two groups, *r* = 0.03, *p* = 0.774 (see Table 1). Inside the groups, the change could be observed on a very small population, as shown by the effects size, placebo *r* = 0.18, *p* = 0.329; and *Asp* = 0.13, *p* = 0.501. An additional three patients, two in the placebo group and one in the *Asp* group, presented an undetectable HIV-1 viral load at the end of intervention. Those outlier patients did not report any antiretroviral medication or the start of HAART.

The assessment of the immune activity through the CD38 expression on the CD8 T-lymphocyte after the pilot RCT did not differ between the groups, *r* = 0.04, *p* = 0.732 (see Table 2). Inside the groups, the T-CD8/CD38 lowered: *Asp*, −1.3 (−5.8%–2.0%), *r* = 0.24, *p* = 0.185; and placebo, −0.9 (−4.17%–1.56%), *r* = 0.18, *p* = 0.327. Furthermore, an immune activation over 40% T-CD8-CD38 present in the study seemed to be related to concomitant symptoms occurring during the intervention (data not shown). Due to the low patient numbers with concomitant symptoms, further relations could not be found.

**Figure 2:**
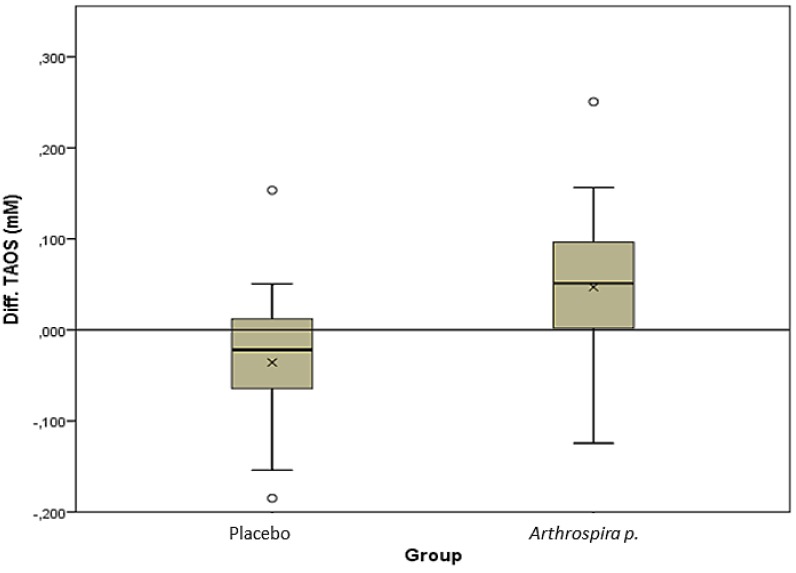
TAOS difference during the RCT between placebo and *Arthrospira platensis*. The box plot represents the median and the IQR. The zero line indicates no change. The crosses represent the means.

The TAOS trends occurring in the two groups were significantly opposite of each other, *r* = 0.51, *p* < 0.001 (see Table 2 and Figure 2). The TAOS increased in the *Asp* group by 56 (1–98) µM, *r* = 0.51, *p* = 0.007; whilst it decreased in the placebo group by −22 (−64–19) µM (*r* = 0.48, *p* = 0.008). Furthermore, in the *Asp* group, the TAOS increase was negatively correlated with the baseline (Rho = −0.436, *p*= 0.02).

Both groups showed a weight increase of 0.65 (−0.30–2.50) kilograms over the three months of intervention: *Asp*, *r* = 0.12, *p* = 0.517; and placebo, *r* = 0.52, *p* = 0.005. The weight difference between the groups after three months was not significant: *r* = 0.21, *p* = 0.105 (see Table 2).

The difference in serum albumin that occurred during the intervention was not significantly different between the groups: *r* = 0.07, *p* = 0.595. An observed slight decrease of the serum albumin concentrations in both groups from 49 (47–52) g/L to 47 (45–50) g/L was found statistically significant (placebo group, *r* = 0.39; *p* = 0.034; and *Asp* group, *r* = 0.42; *p* = 0.026.

**Table 2:** Study variables. TAOS, total antioxidant status in blood serum.

Baseline				Intervention		Difference	
Placebo *n* = 30		*Arthrospira n* = 28		Placebo *n* = 30	*Arthrospira n* = 28	Placebo *n* = 30	*Arthrospira n* = 28
Variables	**Median (IQR)**		**Median (IQR)**	**Median (IQR)**	**Median (IQR)**	**Median (IQR)**	**Median (IQR)**
CD4 (Cells/mm^3^)	462 (413–558)		440 (415–550)	417 (311–486)	406 (320–499)	−52 (−112–−16)	−66 (−111–−20)
Viral load (Log10)	5.6 (5.1–6)		5.3 (4.3–5.8)	5.5 (4.7–5.9)	5.3 (4.5–5.9)	0.0 (−0.4–0 0.2)	0.05 (−0.2–0.4)
CD8/CD38 (%)	25.2 (17.1–37.1)		22.4 (16.2–30.5)	21.2 (15.5–36.3)	24.5 (14.5–29.9)	−0.9 (−4.17–1.56)	−1.3 (−5.8–2.0)
Weight (kg)	56. 8 (54.1–61.7)		57.3 (53.2–62.3)	58.4 (54.9–63.1)	57.8 (52.9–62.5)	0.65 (−0.1–2.9)	0.5 (−0.35–1.65)
TAOS (µM)	357 (298–424)		330 (275–384)	336 (275–373)	387 (320–430)	−22 (−64–19)	56 (1–98)
Albumin (g/L)	49.5 (47–52)		49 (47–51)	47.5 (46–49)	47 (44.5–50)	−1.5 (−4.0–1)	−3.0 (−4–0)
Urea (g/L)	0.19 (0.16–0.22)		0.17 (0.13–0.20)	0.18 (0.15–0.22)	0.18 (0.12–0.22)	0.00 (−0.30–0.04)	0.00 (−0.35–0.03)
Creatinine (mg/dL)	0.7 (0.6–0.8)		0.7 (0.6–0.7)	0.7 (0.6–0.8)	0.75 (0.6–0.85)	0.0 (−0.1–0.1)	0.1 (0.0–0.2)
eGFR (mL/min)	57.7 (50.5–61.1)		59.8 (51.2–72)	56.6 (48.7–70.5)	51.3 (43–67.1)	0.01 (−6.7–4.6)	−7.3 (−17.2–0.9)

Under the intervention with *Asp*, the serum creatinine rose from a baseline of 0.7 (0.6–0.7) mg/dL to 0.75 (0.6–0.85) mg/dL (*r* = 0.59, *p* = 0.002); whilst the placebo group did not show any change in serum creatinine over the intervention (*r* = 0.12, *p* = 0.51). This difference between the two groups after the intervention was significant, *r* = 0.35, *p* = 0.008. The concentration dependence of the eGFR value confirms the slight increase of serum creatinine occurring in the *Asp* group. The differences after the intervention were 0.01 (−6.73–4.62) mL/min for the placebo and −7.31 (−17.2–0.95) mL/min for *Asp*, *r* = 0.32; *p* = 0.016. The urea concentration showed no change between the groups (*r* = 0.13, *p* = 0.326) after the intervention.

## 4. Discussion

This randomized control trial assessed the change of relevant infection outcomes in two groups of female patients with preclinical HIV-infection receiving a dietary supplementation over three months. The intervention supplement differed from the placebo in its content of vitamins, minerals and the bioactive plant molecules present in *Asp*.

In this study, we did not observe any benefit of an intake of five grams of *Asp* per day over three months on the viral load and/or the CD4 T-cells. The groups significantly differed on the antioxidant capacity and slightly in the creatinine serum levels, with increases for both outcomes in the *Asp*-supplemented group. As stated, all changes that occurred on the aforementioned markers per group over the intervention documented by the effect size showed a non-significant benefit for the *Asp*-supplemented group, a lower decrease for CD4 (*Asp*: *r* = 0.61, placebo: *r* = 0.67) and a larger decrease of the immune activation CD8-CD38 (Plac: *r* = 0.18, *Asp*: *r* = 0.24). The lack of a significant difference between the groups on the VL, CD4 and/or CD8-CD38 could be related to the short intervention duration and/or the lack of power. The *Asp* intervention effect on immune variables documented by the effect size and mentioned by the literature may relate to its rich content in polyphenols. A review mentioned an epigenetic mechanism of the immune modulation of polyphenol, which would relate an *Asp* action to a long-term action [35]. Furthermore, the antiretroviral effects of *Asp* described *in vitro* were related to the property of sulfated polysaccharide complexes with Ca^2+^ ions acting on the virus docking [27,36], but they could not be confirmed in the present human study.

A connection between nutrition and infection is related to oxidative stress and the antioxidant status [37]. The measurement of the TAOS capacity is reported to provide an integrated index of antioxidant potential, regrouping enzyme and dietary antioxidants, as opposed to a simple summation of measurable antioxidants [38]. As noted in the placebo group, the TAOS decrease (*r* = 0.48, *p* = 0.008) reflects the progression of infection [39]. In contrast, *Asp* had a significantly increased effect on the TAOS (*r* = 0.51, *p* = 0.007). Defining the *Asp* effect, the significant negative correlation between the changes occurring during the *Asp* intervention and the TAOS baseline value (Rho = −0.436, *p* = 0.02) could be interpreted as a rehabilitation of patients with initially low TAOS.

Oxidative stress can impact HIV proliferation, because the gene regulator, NF-κB, has been reported to enhance the gene expression of the virus [40]. The NF-κB pathway is upregulated via oxidative stress [40,41]. No relation between the increase of antioxidant capacity and a lowering of the viral particle count in the blood could be detected in this study. The viral load level of 300,000 copies/mL is considered high and related to an acute infection progress, marking the end of the latent period [34]. The high level of viral particles at baseline could mask the *Asp* rehabilitation effect on the antioxidative capacity and its impact on the viral load.

The intervention did not aim to increase the quantity by a supplementation of 3.1 g of protein per day, but rather, to ameliorate the quality with all essential amino acids present in both products. The results have shown a similar positive impact on weight for both supplemented products. The significant increase for the placebo preparation would support the assumption of a stimulation of anabolic action by the protein content (*r* = 0.52, *p* = 0.005). The stable weight for the *Asp* preparation could be due to a functional catabolic implementation of *Asp* activity (*r* = 0.12, *p* = 0.517). The lack of a significant difference between the two products expresses the equal mixture of the *Asp* product and the placebo in terms of energy and protein gain (*r* = 0.21, *p* = 0.105).

Furthermore, *Asp* was found to be associated with a significant increase of 0.1 mg/dL serum creatinine (*r* = 0.59, *p* = 0.002), still far below the clinically relevant mark of 1.5 mg/dL. Dietary protein intakes, a change in the filtration capacity of the kidneys or intense physical activity are factors influencing serum creatinine. The increase of the serum creatinine was also reported in a study supplementing 10 g of *Asp* daily in the Central African Republic [42], but was not associated with an increase of the serum urea. Therefore, it can be concluded that the additional amount of five grams of protein-rich supplement per day has no effect on *N*-metabolism.

The outbreak of concomitant events is an indicator of the patients’ vulnerability and commonly indicates the need for starting HAART. As from the start patients were excluded who started HAART or needed relevant medication for infections and as the study patients were in the preclinical stage of the diseases, the rate of registered opportunistic infections was low: in the placebo group, three opportunistic infections occurred and there was no related exclusion. In the *A. platensis* group, there were five exclusions due to the starting of HAART and no occurrence of opportunistic infections. 

There is still a paucity of clinically controlled trials to evaluate *Asp* effects on humans and, particularly, on HIV patients. Recent studies have started to fill the gap between laboratory research and human application. A recent trial documented an improvement of anemia status and immunosenescence in healthy people after *Asp* supplementation [43]. An HIV-related trial has shown good nutritional rehabilitation effects by the consumption of *Asp* mixed into the rehabilitation food of undernourished children in Burkina Faso [44]. Another trial on HAART patients taking *Asp* has shown the amelioration of the insulin sensitivity compared to patients supplemented with soy beans [45]. One investigation could show that the combination of *Asp* and HAART resulted in a significant gain of a 99 CD4 cells/mL count compared to a gain of 46 CD4 cells/mL for soy and HAART over a study period of three months [46].

Ingestion of *A. platensis* in other forms, such as enriched *Asp* extracts [47], or in larger amounts of raw powder mixed into already cooked food could be an appropriate way to enhance the beneficial effect of *Asp* for HIV-infected patients.

Furthermore, the origin of the supplement has to be clearly defined. The culture of *Asp* can be artisanal, semi-industrial, industrial or naturally harvested. The growing condition, the quality of the water and correct drying are major factors influencing the safety of the product, as well as its nutrient content. Therefore, some results of this study may not be representative for *A. platensis* products derived from algae grown in Lake Chad or other freshwater locations. The growing conditions of the algae have to be considered in terms of the enrichment of the content of nutrients, e.g., calcium polysaccharide and lipids, as well as bioactive molecules, such as phycocyanins. 

With the non-significant observations of a difference in concomitant events and the time of progression of the disease resulting in the starting of HAART, it could be useful for future studies to also assess the properties of *Spirulina* regarding the time from seroconversion to the initiation of HAART.

## 5. Conclusions

In the context of the present study, *Arthrospira platensis* is a food rich in nutrients and bioactive molecules that can be grown locally. The consumption in the form of 10 capsules of dry powder, up to five grams a day, showed a non-significant clinical effect and no immunological activity for pre-HAART HIV-infected women over three months. Viral load and CD4 cell count were unaffected. However, the intervention seemed to reduce the incidence of concomitant events, as well as opportunistic infections and showed a positive effect on weight stabilization.

Although the number of cases in this study was low, an effect of *Asp* on the antioxidant status could be documented in HAART-naive HIV patients. The use of *Asp* for antiretroviral activity should be looked at carefully, since we did not generate data supporting such an effect in the present study. However, *Asp* can be recommended as a food supplement capable of reinforcing the body’s antioxidative status.
